# Tailoring the expression of Xyr1 leads to efficient production of lignocellulolytic enzymes in *Trichoderma reesei* for improved saccharification of corncob residues

**DOI:** 10.1186/s13068-022-02240-9

**Published:** 2022-12-17

**Authors:** Linjing Shen, Aiqin Yan, Yifan Wang, Yubo Wang, Hong Liu, Yaohua Zhong

**Affiliations:** State Key Laboratory of Microbial Technology, Institute of Microbial Technology, Shandong University, Qingdao, 266237 People’s Republic of China

**Keywords:** *Trichoderma reesei*, Xyr1, Transcription activator, Cellulase, Saccharification

## Abstract

**Background:**

The filamentous fungus *Trichoderma reesei* is extensively used for the industrial-scale cellulase production. It has been well known that the transcription factor Xyr1 plays an important role in the regulatory network controlling cellulase gene expression. However, the role of Xyr1 in the regulation of cellulase expression has not been comprehensively elucidated, which hinders further improvement of lignocellulolytic enzyme production.

**Results:**

Here, the expression dosage of *xyr1* was tailored in *T. reesei* by differentially overexpressing the *xyr1* gene under the control of three strong promoters (P*egl2*, P*cbh1,* and P*cdna1*), and the transcript abundance of *xyr1* was elevated 5.8-, 12.6-, and 47.2-fold, respectively. We found expression of cellulase genes was significantly increased in the P*egl2*-driven *xyr1* overexpression strain QE2X, whereas relatively low in the P*cbh1*- and P*cdna1*-driven overexpression strains. We also found that the P*egl2*-driven overexpression of *xyr1* caused a more significant opening of chromatin in the core promoter region of the prominent cellulase genes. Furthermore, the cellulase activity showed a 3.2-fold increase in the strain QE2X, while insignificant improvement in the P*cbh1*- and P*cdna1*-driven strains. Finally, the saccharification efficiency toward acid-pretreated corncob residues containing high-content lignin by the crude enzyme from QE2X was increased by 57.2% compared to that from the parental strain. Moreover, LC–MS/MS and RT-qPCR analysis revealed that expression of accessory proteins (Cip1, Cip2, Swo1, and LPMOs) was greatly improved in QE2X, which partly explained the promoting effect of the P*egl2*-driven overexpression on enzymatic hydrolysis of lignocellulose biomass.

**Conclusions:**

Our results underpin that the precise tailoring expression of *xyr1* is essential for highly efficient cellulase synthesis, which provide new insights into the role of Xyr1 in regulating cellulase expression in *T. reesei*. Moreover, these results also provides a prospective strategy for strain improvement to enhance the lignocellulolytic enzyme production for use in biorefinery applications.

**Supplementary Information:**

The online version contains supplementary material available at 10.1186/s13068-022-02240-9.

## Introduction

Lignocellulosic biomass is an abundantly available, inexpensive organic resource on the earth, which is basically composed of cellulose, hemicellulose, and lignin [1]. Biological degradation of lignocellulose biomass by lignocellulolytic enzymes into fermentable sugars for the production of biofuels and other biochemicals has received extensive attention [2, 3]. However, the high cost of cellulase production, which is the major barrier for practical applications, limits the development of an efficient and economically viable lignocellulosic feedstock based biorefinery [4].

The filamentous fungus *Trichoderma reesei* encodes a wide repertoire of cellulase, hemicellulase and accessory proteins to degrade cellulose and other biomass components, which makes it be extensively employed in the industrial cellulase production [5]. The cellulases secreted from *T. reesei* mainly include three enzyme components, namely, cellobiohydrolases (CBHs, EC 3.2.1.91), endoglucanases (EGs, EC3.2.1.4), and β-glucosidase (exactly BGL1, EC 3.2.1.21) [6]. In general, CBHs and EGs act cooperatively to degrade cellulose into cellobiose, and BGL1 ultimately hydrolyzes the oligosaccharide into glucose [6]. The three components work synergistically to accomplish a complete deconstruction of cellulose [7]. Optimizing the composition of cellulase complexes by genetic engineering to strengthen the synergism has been proved to be practical [8, 9], among which the overexpression of BGL or EGs is a powerful strategy for enhancing the synergistic effect [10–12]. Recently, Qian et al*.* demonstrated that the double overexpression of EG2 and BGL1 can significantly ameliorate the performance on the cellulase yield and saccharification efficiency in *T. reesei* [13]. Moreover, it is known that cellulase gene expression is stringently controlled by transcriptional regulatory networks in *T. reesei* [14]. Therefore, engineering the transcriptional factors can improve the entire cellulase system, which has been considered to be an effective way for enhancing cellulase production.

The energy-efficient production of cellulase in fungi is strictly regulated by a suit of transcription factors [15]. So far, several transcription factors involved in the modulation of cellulase gene expression have been identified, including at least five transcription activators (Xyr1, Ace2, Ace3, Vib1, and the Hap/2/3/5 complex) and four repressors (Cre1, Ace1, Rce1, and Ctf1) [16–19]. Other transcription factors, such as BglR, Pac1, and Crz1, have also been identified to regulate the biosynthesis of cellulase [20–22]. Especially, Xyr1 is the transcriptional activator modulating the expression of cellulase and hemicellulase genes in *T. reesei* [24]. Overexpression of *xyr1* can markedly enhance cellulase production in *T. reesei* [26, 28]. In addition, Xyr1 also seems to be involved in the glucose repression of cellulase mediated by the carbon catabolite repressor Cre1, since its constitutive expression being able to reduce this repressive effect [4, 25, 26]. It has been reported that the expression levels of the main cellulase genes (*cbh1* and *cbh2*) appeared to be strictly dependent on the expression level of *xyr1* [25]. Zheng et al. overexpressed the *xyr1* gene under the control of the copper repressible promoter P*tcu1* and the transcript level was improved by approximately twofold on Avicel [27]. However, it seemed that the efficiency of the *tcu1* promoter in enhancing transcription of the major cellulase gene *cbh1* remains lower compared to the strong pyruvate decarboxylase gene (*pdc*) promoter, with the transcript level of *xyr1* was increased by 16-fold under the similar condition [27, 28]. As noted above, tuning the expression dosage of *xyr1* may have profound effects on cellulase expression in *T. reesei*, which requires more systematical and in-depth investigation.

In the present study, the expression of *xyr1* was tailored with three differential strong promoters (P*egl2*, P*cbh1,* and P*cdna1*) in *T. reesei*. We found that the overexpression of *xyr1* driven by P*egl2*, not that driven by the stronger promoter P*cbh1* or P*cdna1*, leads to more significantly enhanced cellulase production. Meanwhile, it also resulted in a significant opening of chromatin in the core promoter regions of cellulase genes, which was accompanied by the activation of the corresponding gene expression. Furthermore, the overexpression of *xyr1* with P*egl2* also remarkably improved the levels of accessory proteins involved in lignocellulose degradation and thus promoted the saccharification efficiency toward acid-pretreated corncob residues.

## Results

### Overexpression of the xyr1 gene using the promoters with different strengths in T. reesei

**Fig. 1:**
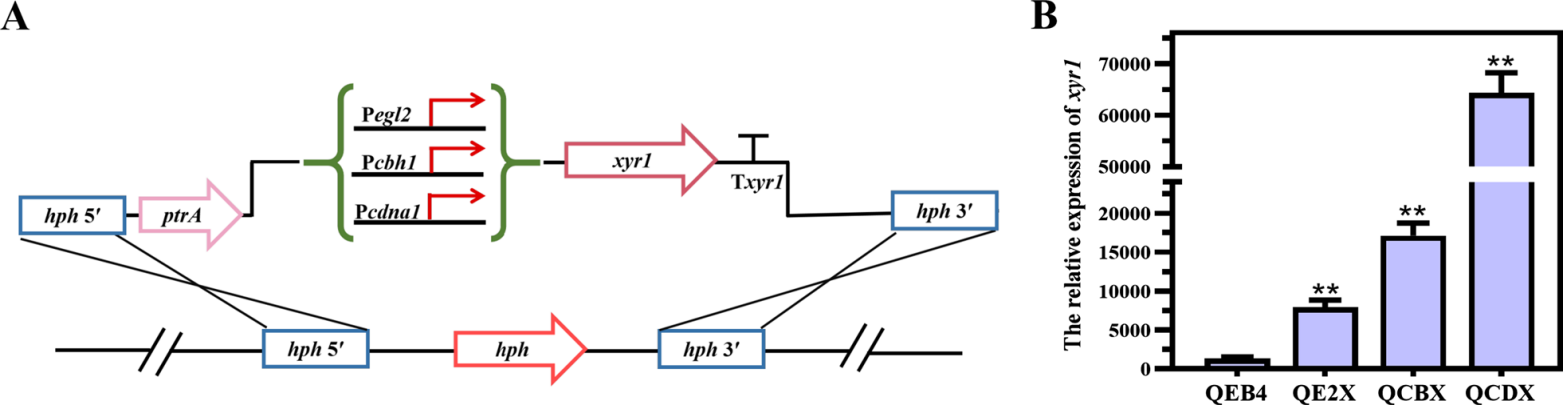
Construction and validation of the *xyr1* overexpression strains. **A** Schematic diagrams of the expression cassettes used for differential overexpression of the *xyr1* gene. The overexpression of *xyr1* was controlled by three different promoters (P*egl2*, P*cbh1,* and P*cdna1*), respectively. **B** Transcript levels of *xyr1* which was determined by RT-qPCR in QEB4 and three independent transformants of each *xyr1* overexpression strain (QE2X, QCBX, and QCDX). Strains were cultivated at 30 ℃ and 200 rpm in flasks using the CPM medium with 2% (w/v) Avicel as the sole carbon source for 72 h. The transcript level was normalized to that of *actin* (*t*-test, **p < 0.01). Results are means of three biological replicates and error bars indicate ± SD

To systematically investigate the effect of differential expression of *xyr1* on cellulase production in *T. reesei*, the *xyr1* overexpression cassettes driven by the cellulose-inducible promoters P*egl2*, P*cbh1,* and the strong constitutive promoter P*cdna1* were constructed and precisely integrated by homologous recombination into the *hph* locus of *T. reesei* QEB4, respectively (Fig. 1A). The strains harboring the *xyr1* gene driven by the promoters P*egl2*, P*cbh1,* and P*cdna1* were designated QE2X, QCBX, and QCDX, respectively. The integration of the *xyr1* gene into the genome of *T. reesei* QEB4 was verified by PCR (data not shown). Furthermore, the transcript levels of *xyr1* were analyzed by RT-qPCR (Fig. 1B). It was found that *xyr1* was significantly overexpressed in the strains, with the maximum transcript levels in the QCDX strain, followed by QCBX and QE2X, which were 47.2-, 12.6-, and 5.8-fold higher than the parental strain QEB4, respectively.

Next, the three *xyr1* overexpression strains QE2X, QCBX, and QCDX were cultured on minimal medium (MM) plates containing glucose, glycerol, and lactose as the carbon source, respectively, as well as on PDA plates to investigate the effect of differential *xyr1* overexpression on mycelial phenotypes of *T. reesei*. As shown in Additional file 1: Fig. S1, the colonies of the recombinant strains extended normally and grew at a rate similar to that of the parental strain, indicating that different dosage levels of *xyr1* had little effect on mycelial growth of *T. reesei*.

**Fig. 2:**
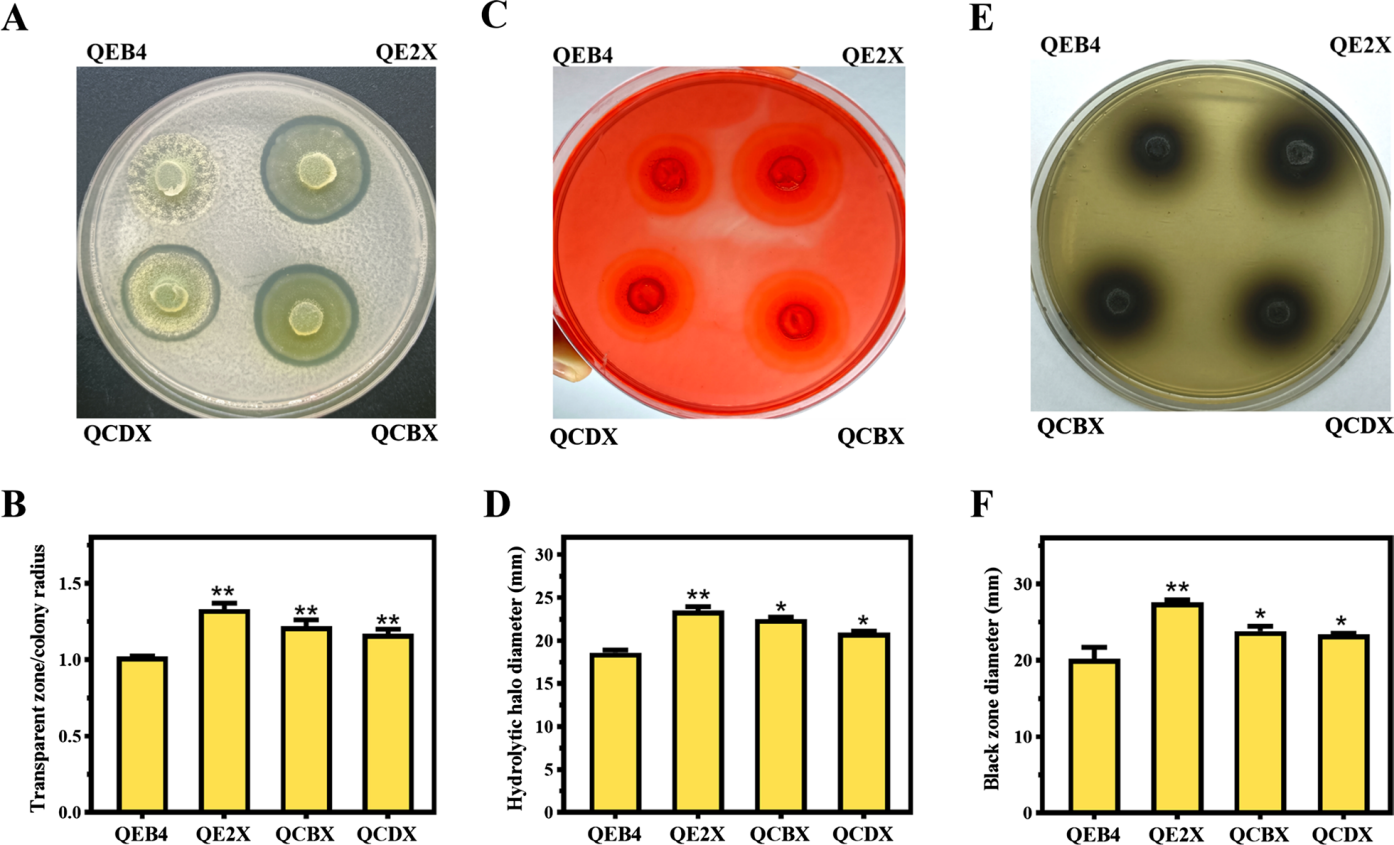
Detection of the cellulase secretion by *T. reesei* QEB4 and the *xyr1* overexpression strains in different culture plates. The strains were assessed on the MCC plate (**A**), the CMC plate (**C**), and the CMC-esculin plate (**E**) to evaluate the secretion capacity of total cellulase, EGs and BGL, respectively. **B** The ratios between the diameter of the transparent zones and the colonies on MCC plate. **D** The hydrolytic halo diameters of the strains on CMC-Na plate. **F** The black zone diameters of the strains on CMC-esculin plate. *p < 0.05; **p < 0.01 (*t*-test). Results are means of three biological replicates and error bars indicate ± SD

Thereafter, the recombinant strains were inoculated on microcrystalline cellulose (MCC), CMC, and CMC-esculin plates to assess the cellulase secretion capacity (Fig. 2). These strains displayed wider hydrolytic haloes or black zones around the colonies relative to the parental strain, suggesting that the cellulase secretion ability was enhanced to different extents. Especially, the cellulase secretion in QE2X strain with the moderately strong promoter P*egl2* was far greater than that in the other two strains with stronger promoters (P*cbh1* and P*cdna1*) on the plates, implying that there may be a dose-dependent correlation between the expression level of *xyr1* and the secretion of cellulase in *T. reesei*.

### Influences of differential overexpression of xyr1 on the transcriptional profile of cellulase genes

**Fig. 3:**
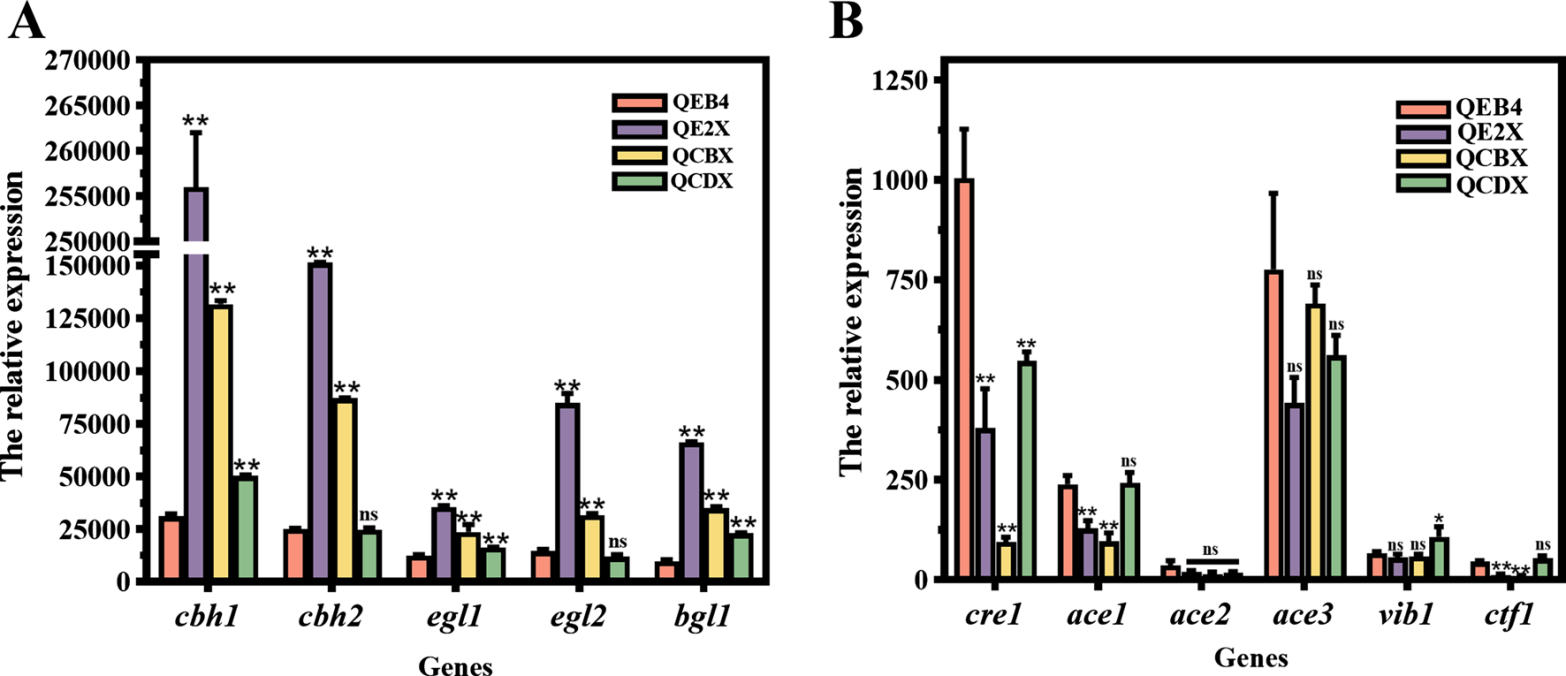
Transcript analysis by RT-qPCR for genes encoding cellulases and regulators. Genes encoding cellulases (**A**) and transcription factors (**B**) were analyzed in the *xyr1* overexpression strains with *T. reesei* QEB4 as the control. Strains were cultivated at 30℃ and 200 rpm in flasks using the CPM medium with 2% (*w/v*) Avicel as the sole carbon source for 72 h. The transcript levels of genes were normalized to that of *actin* (*t*-test, *p < 0.05, **p < 0.01, ns = not significant). Results are means of three biological replicates and error bars indicate ± SD

It is known that transcription of the cellulase-encoding genes are modulated by the transcriptional activator Xyr1 [23]. Here, the transcript levels of five main cellulase-encoding genes *cbh1*, *cbh2*, *egl1*, *egl2,* and *bgl1* were measured by RT-qPCR in the *xyr1* overexpression strains (Fig. 3A). It was found that the transcript quantities of the five cellulase genes in the QE2X strain with the lowest degree of *xyr1* overexpression (5.8-fold) were 7.3-, 5.0-, 1.4-, 4.8-, and 5.8-fold higher than those of the parental strain, respectively. However, those in the QCBX strain with the stronger *xyr1* overexpression (12.6-fold) were only slightly increased by 3.2-, 2.5-, 0.9-, 1.2-, and 2.6-fold, respectively. Unexpectedly, the transcript levels of these cellulase genes in the QCDX strain with the highest degree of *xyr1* overexpression (47.2-fold) were not significantly improved. Taken together, these results demonstrate that cellulase gene transcription is precisely regulated by the *xyr1* transcript abundance, i.e., the moderately strong overexpression of *xyr1* with the P*egl2* promoter, but not the stronger overexpression with the P*cbh1* or P*cdna1* promoter, could lead to more pronounced improvement in cellulase expression.

Furthermore, transcript analysis of several transcription factors involved in cellulase expression were performed (Fig. 3B). The positive transcriptional regulators-encoding genes *ace2*, *ace3*, and *vib1* were virtually unchanged in all the recombinant strains. However, the negative transcriptional regulator-encoding genes *cre1*, *ace1,* and *ctf1* were greatly decreased in both QE2X and QCBX. As for the QCDX strain, the expression of *ace1* and *ctf1* showed no significant changes, while the carbon catabolite repressor-encoding gene *cre1* was downregulated. These findings suggest that overexpression of *xyr1* probably resulted in downregulation of the negative regulators, thus facilitating improvement of the expression of cellulase genes.

### Differential overexpression of xyr1 regulated the chromatin accessibility of cellulase genes and the native xyr1 gene

**Fig. 4:**
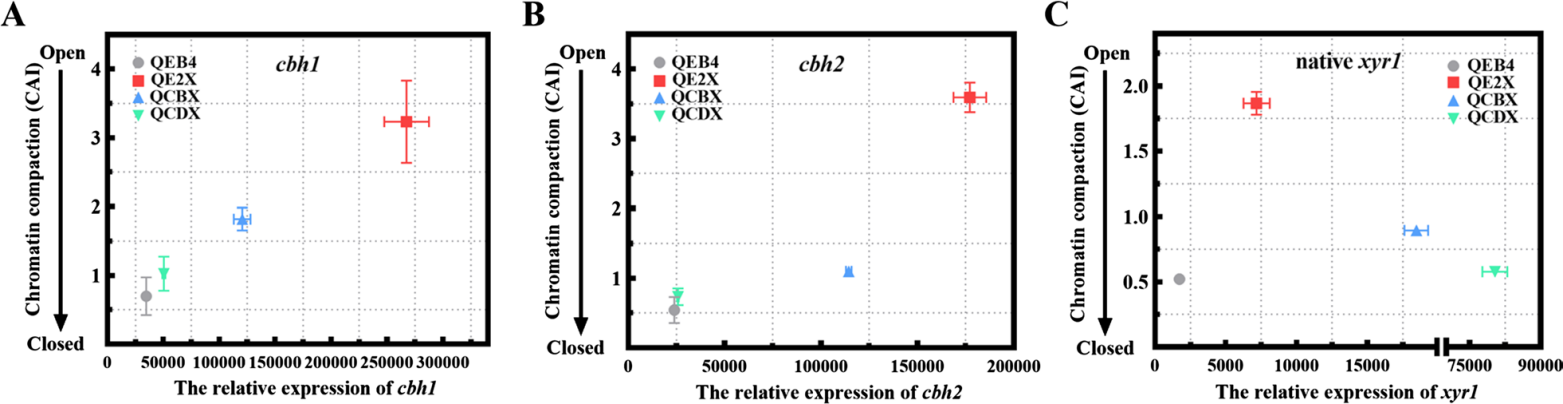
Transcript and CHART analysis of *T. reesei* QEB4 and the *xyr1* overexpression strains. The genes of *cbh1* (**A**), *cbh2* (**B**), and the native *xyr1* (**C**) were determined by RT-qPCR and CHART-PCR, respectively. The strains were cultured in CPM with 2% (w/v) Avicel for 48 h. All data were normalized to the reference gene *actin.* The transcript levels are depicted on the x-axis and the chromatin accessibility indices (CAI) are plotted on the y-axis. Results are means of three biological replicates and error bars indicate ± SD

Eukaryotic gene expression is often accompanied by chromatin remodeling [29]. It was reported that the deletion of *xyr1* in *T. reesei* resulted in the loss of expression of the cellulase genes *cbh1* and *cbh2* and the reduction in chromatin accessibility of these genes [30]. To gain further insight into the impact of *xyr1* dosage, the chromatin packing of the core promoter region of *cbh1* and *cbh2* in the *xyr1*-overexpressing strains was analyzed by CHART-PCR (Fig. 4A, B). In all the recombinant strains, the chromatin accessibility index (CAI) in the promoter region of *cbh1* and *cbh2* was improved to varying degree which was corresponded with the high expression levels. This suggests that the *xyr1* overexpression can lead to more open chromatin status of cellulase genes. As expected, remarkable chromatin opening of *cbh1* and *cbh2* was detected in QE2X, indicating that the *egl2* promoter-driven *xyr1* overexpression is more efficacious in promoting the chromatin accessibility of cellulase-encoding genes than the stronger *cbh1/cdna1* promoter-driven overexpression.

It was also known that the expression level of *xyr1* may correlate with the degree of chromatin opening in its own promoter [31]. Hence, the chromatin packing of the native *xyr1* core promoter region in the *xyr1*-overexpressing strains was analyzed (Fig. 4C). More open chromatin status was found in all the recombinant strains, with the most significant chromatin opening in QE2X. These results further corroborate the view that the dosage level of *xyr1* differentially influenced the chromatin status of its own promoter.

### Differential overexpression of xyr1 induced the unfolded protein response in the endoplasmic reticulum

**Fig. 5:**
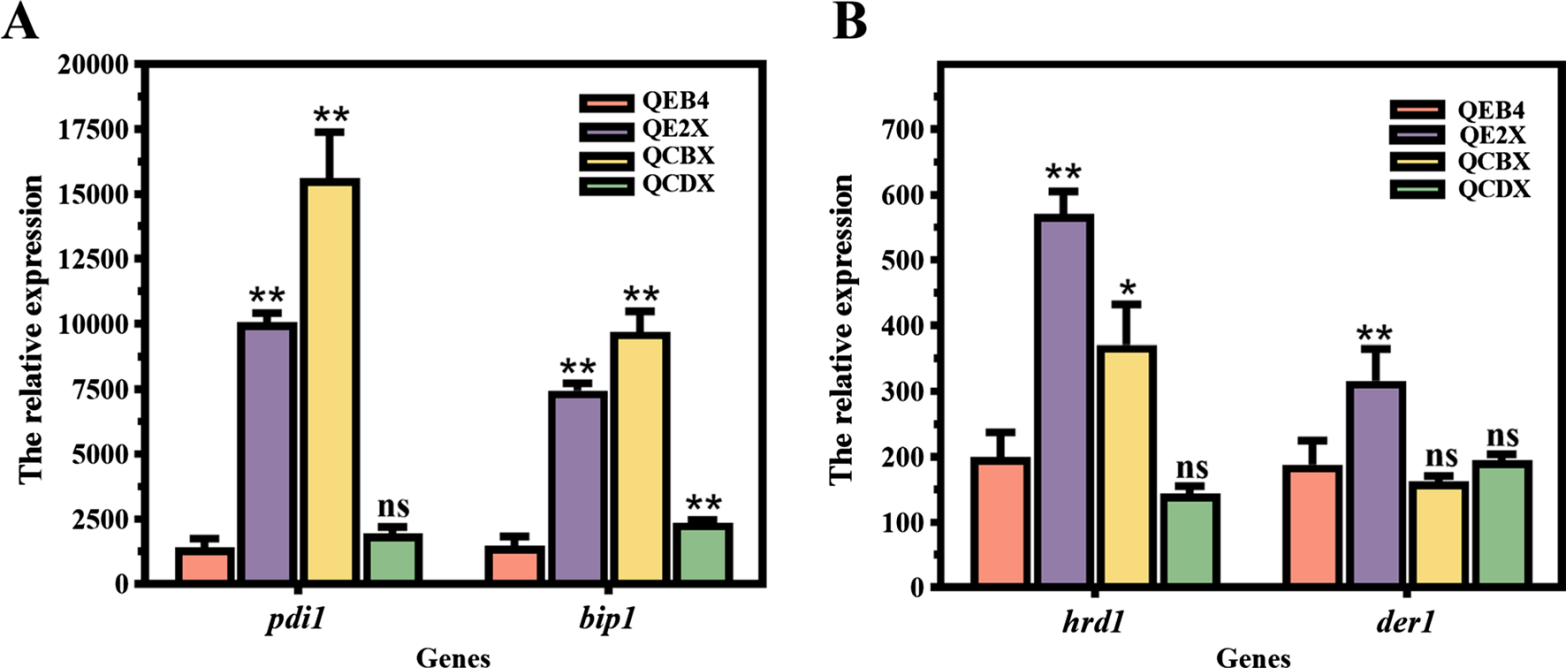
Transcript analysis of the genes encoding the ER-associated components by RT-qPCR. The UPR- (**A**) and ERAD- (**B**) related genes were analyzed in the *xyr1* overexpression strains with *T. reesei* QEB4 as the control. Strains were cultivated at 30 ℃ and 200 rpm in flasks using CPM with 2% (w/v) Avicel as the sole carbon source for 72 h. The transcript levels of genes were normalized to that of *actin* (*t*-test, *p < 0.05, **p < 0.01, ns = not significant). Results are means of three biological replicates and error bars indicate ± SD

As the major secreted proteins in *T. reesei*, the newly synthesized cellulase enzymes should enter the endoplasmic reticulum (ER) secretory pathway where they fold into the correct three-dimensional structure with the assistance of resident ER folding factors for secretion, whereas those that misfold would be efficiently degraded by the ER-associated degradation (ERAD) [32]. Meanwhile, the accumulation of unfolded proteins in the ER can cause ER stress, which elicits the unfolded protein response (UPR), a mechanism to upregulate UPR-regulated genes involved in protein folding, ERAD, and others for the restoration of ER homeostasis [33]. To investigate the effect of differential expression of *xyr1* on the secretory pathway, the expression of genes encoding key components involved in protein folding (*pdi1* and *bip1*) and the ERAD pathway (*hrd1* and *der1*) was determined in the *xyr1*-overexpressing strains. As shown in Fig. 5, all tested genes (*pdi1*, *bip1*, *hrd1,* and *der1*) were significantly upregulated in QE2X, while only the folding factor genes (*pdi1* and *bip1*) were upregulated in QCBX and QCDX. These above results demonstrate that the *xyr1* overexpression could induce the UPR in the ER. In particular, the strong ER stress evoked in QE2X resulted in significant activation of the ERAD pathway, indicating a large amount of cellulase has been accumulated in the ER, i.e., saturation of the secretory pathway may be reached in this strain.

### Pegl2-driven xyr1 overexpression greatly enhanced cellulase production in T. reesei

**Fig. 6:**
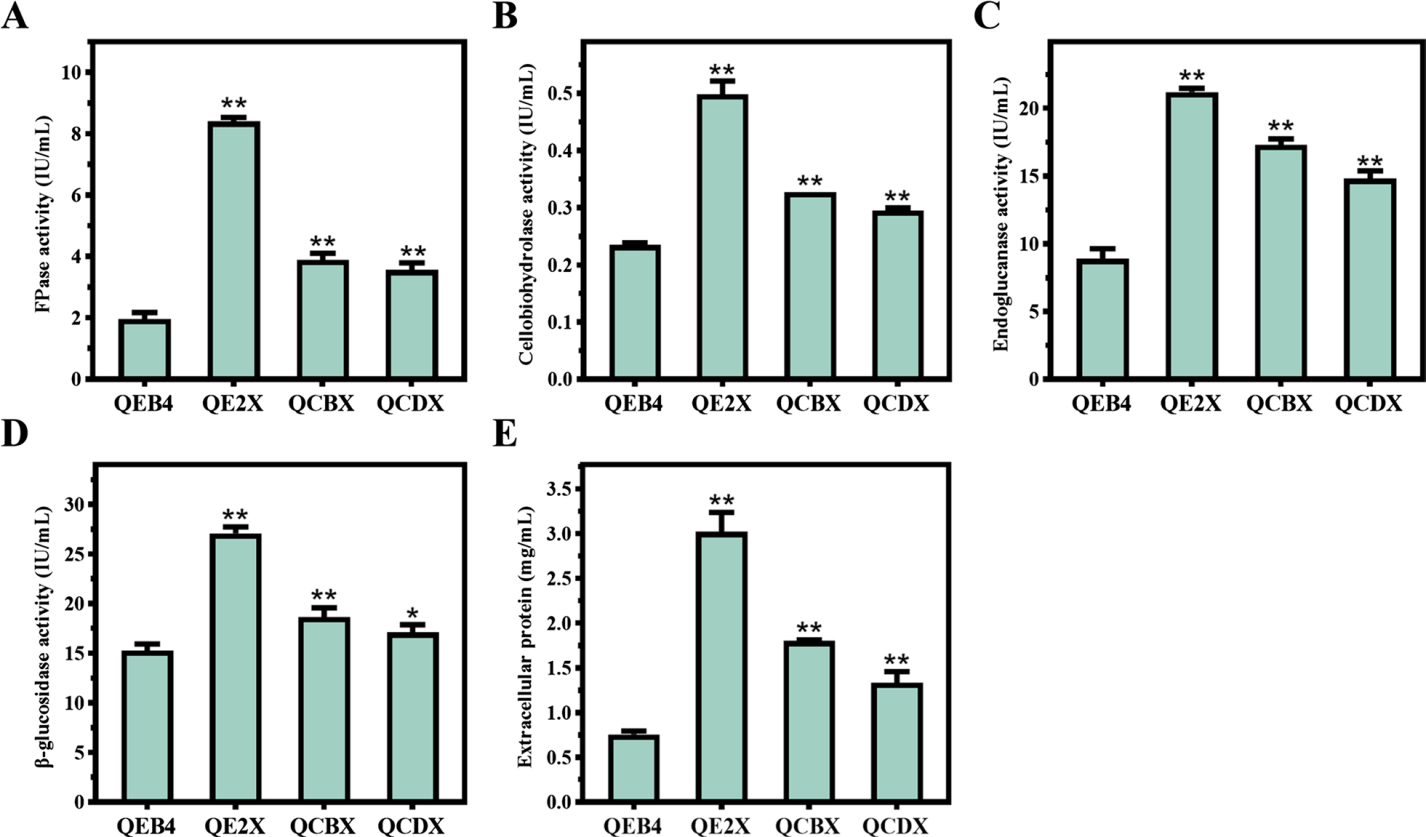
Cellulase activities and total extracellular proteins of *T. reesei* QEB4 and the *xyr1* overexpression strains. **A** FPase activity (FPA), **B** cellobiohydrolase (CBH) activity, **C** endoglucanase (EG) activity, **D** β-glucosidase (BGL) activity, and **E** the total extracellular protein of the fermentation supernatant from QEB4 and three recombinant strains cultured on 2% (w/v) Avicel for 7 days. *p < 0.05; **p < 0.01, ns = not significant (*t*-test). Results are means of three biological replicates and error bars indicate ± SD

To investigate whether the cellulase production levels were improved in the *xyr1* overexpression strains, the activities of cellulase in the fermentation broths were determined under Avicel-inducing conditions. The total cellulase activity of QE2X reached 8.4 IU/mL, 3.2-fold higher than that of the parental strain (2.0 IU/mL), using filter paper activity (FPA) as a characterization method, while the FPA in QCBX and QCDX was only elevated 0.9- and 0.8-fold compared to that of the parental strain, respectively (Fig. 6A). Consistently, QE2X also displayed a significant enhancement in the activities of the cellulase components, with 2.1-, 2.4-, and 1.8-fold increase in CBHs, EGs, and BGL activities, respectively. However, QCBX and QCDX only demonstrated 1.4- and 1.3-fold improvement in CBH activity, 1.9- and 1.7-fold improvement in EG activity as well as 1.2- and 1.1-fold improvement in BGL activity, respectively (Fig. 6B–D). In addition, the concentration of extracellular protein in QE2X reached 3.0 mg/mL, much higher than that in QCBX and QCDX (Fig. 6E). Taken together, these results indicate that the *egl2* promoter-driven *xyr1* overexpression can significantly enhance cellulase production in *T. reesei.*

### Pegl2-driven overexpression of xyr1 optimized the lignocellulolytic enzyme system for saccharification of corncob residues

**Fig. 7:**
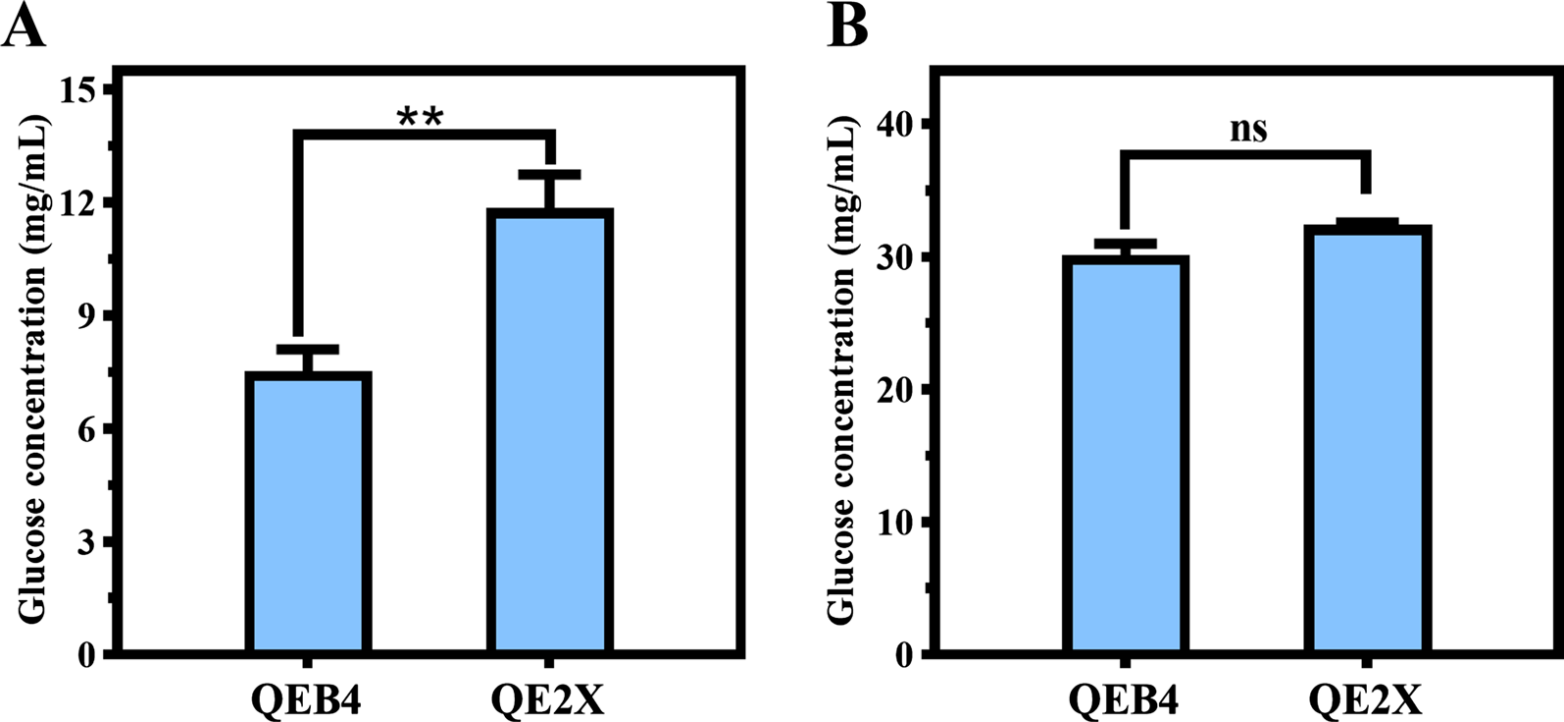
Saccharification of different pretreated corncob residues by *T. reesei* QEB4 and QE2X. Saccharification of acid-pretreated corncob residue (**A**) and delignified corncob residue (**B**) with the same FPA dosage. **p < 0.01, ns = not significant (*t*-test). Results are means of 3 biological replicates and error bars indicate ± SD

Among the three *xyr1* overexpression strains, the P*egl2*-driven overexpression strain QE2X showed the highest cellulase activity, and hence, it was selected for subsequent saccharification. Two differently pretreated corncob residues, acid-pretreated (ACR) and delignified (DCR) corncob residues, were utilized as substrates to be saccharified with the enzyme preparations produced by QE2X, with the parental strain QEB4 as control. As shown in Fig. 7, the glucose release from the QE2X enzyme preparation in the saccharification of ACR was 11.9 mg/mL, which was 57.2% higher than that from QEB4. However, no significant difference was observed in the saccharification of DCR between those from QE2X and QEB4. Since the difference in composition between the two corncob substrates is that ACR contains higher levels of lignin than DCR, these results demonstrate that the P*egl2*-driven overexpression of *xyr1* can optimize the lignocellulolytic enzyme system for saccharification of the high lignin-containing lignocellulosic substrates.

**Fig. 8:**
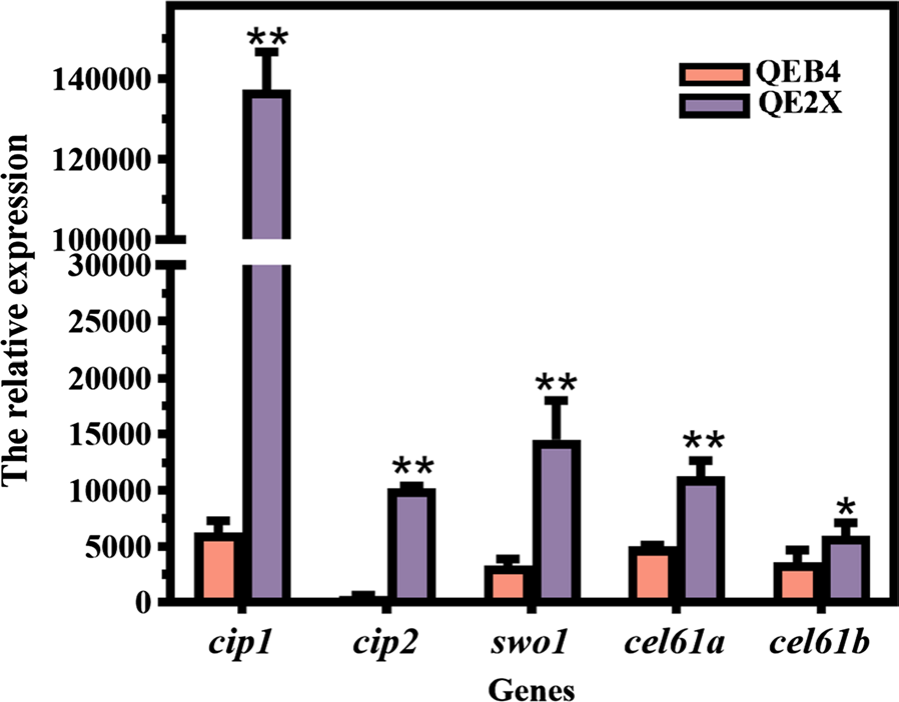
Transcript analysis for genes encoding accessory proteins of *T. reesei* QEB4 and QE2X by RT-qPCR. The transcript levels of genes were normalized to that of *actin* (*t*-test, *p < 0.05, **p < 0.01, ns = not significant). Results are means of three biological replicates and error bars indicate ± SD

It is known that accessory proteins, such as glucuronoyl esterase (Cip1 and Cip2), swollenin (Swo1), and AA9 lytic polysaccharide monooxygenases (LPMOs), play important roles in enhancing lignocellulose degradation [34]. Especially, Cip1 can reduce the non-productive adsorption of lignin by cellulase and Cip2 acts in the cleavage of hemicellulose-lignin crosslinks [35, 36]. Ma et al. demonstrated by RNA sequencing in *T. reesei* that Xyr1 could stimulate the expression of those accessory protein-encoding genes [37]. Thus, the transcript levels of the above accessory protein-encoding genes were firstly examined. The transcript levels of *cip1* and *cip2* in QE2X were improved significantly, which were 21- and 18-fold higher than those in the parental strain QEB4, respectively (Fig. 8). Furthermore, the swollenin-encoding gene *swo1* and two LPMO-encoding genes *cel61a* and *cel61b* were about 3.4-, 1.3-, and 0.7-fold higher in QE2X than those in QEB4, respectively (Fig. 8). Meanwhile, the LC–MS/MS analysis of the secretome of *T. reesei* QE2X and QEB4 was carried out. It was found that, besides the increased amounts of cellulases, the PSMs of all the above-mentioned accessory proteins in QE2X were also higher than those in QEB4 when equal protein was used for evaluation (Table 1), suggesting more accessory proteins were present in the enzyme preparations of QE2X. These results indicate that the P*egl2*-driven overexpression of *xyr1* can optimize the lignocellulolytic enzyme system of *T. reesei* by improving the expression of those accessory proteins, which is beneficial for efficient degradation of lignocellulosic biomass.

**Table 1:** Identified accessory proteins in the 7d fermentation supernatants of *T. reesei* by LC–MS/MS

Protein ID	Name	PSMs	
		*T. reesei* QEB4	*T. reesei* QE2X
73638	Cip1	31	63
123940	Cip2	20	37
123992	Swo1	33	36
73643	Cel61a	7	13
120961	Cel61b	3	7

## Discussion

In *T. reesei*, genetic engineering of transcriptional regulators has been used as a powerful tool for enhancing cellulase production. Xyr1 is the key transcriptional activator of lignocellulolytic genes, whose overexpression can improve cellulase yields under both inducing and non-inducing conditions [26]. However, the effect of *xyr1* dosage regulation on cellulase expression has not yet been elucidated. Herein, the expression of *xyr1* was tailored under promoters of different strengths and we found that the *xyr1* overexpression with the P*egl2* promoter resulted in a substantial improvement in cellulase production and saccharification efficiency.

In previous studies, the promoter P*pdc* was chosen to drive *xyr1* expression for the construction of cellulase hyperproducing strain [4, 28, 38]. In addition, the promoter P*tcu1* was also used to the expression of *xyr1*, and the resultant strain could achieve full cellulase expression [27]. By roughly comparing the effects of the two promoters, we speculate that the overexpression of *xyr1* driven by the stronger promoter may have a more pronounced influence on cellulase production. However, this does not imply that the maximization of cellulases can be achieved by extremely strong overexpression of *xyr1*. Bao et al. found that the moderate overexpression of Sec16 was more potent to increase protein secretion in *Saccharomyces cerevisiae* than the strong overexpression [39]. Thus, uncertain relationships between the degree of *xyr1* overexpression and cellulase gene expression in *T. reesei* limit the potential for cellulase production to some extent. To further determine whether the effect of *xyr1* overexpression depends on its dosage, we selected three strong promoters for tailoring the expression of *xyr1*: the main cellulase-encoding genes promoters P*egl2* and P*cbh1*, as well as the strong constitutive promoter P*cdna1.* Among the *xyr1* overexpression strains, the strain QE2X with the *egl2* promoter exhibited the highest cellulase production, which was consistent with the significantly enhanced cellulase secretion capacity and cellulase gene transcription (Figs. 2, 3A and 6). These findings further suggest that the robust upregulation of cellulase genes caused by the P*egl2*-driven overexpression of *xyr1* result in the outstanding cellulase production observed in the QE2X strain.

It has been reported that the level of *xyr1* transcription tightly regulate the main cellulase genes expression [25]. However, our results showed that the P*egl2*-driven *xyr1* overexpression (5.8-fold) markedly improved both the transcription and chromatin accessibility of cellulase genes, while the effects of the *xyr1* overexpression driven by the *cbh1* and *cdna1* promoters (12.6- and 47.2-fold) were not particularly remarkable (Figs. 3A, 4A, B). It has been reported that upon cellulase induction, Xyr1 is synthesized and rapidly imports into the nucleus in order to regulate cellulase gene expression [40]. In order to investigate the effect of differential *xyr1* overexpression on the Xyr1 protein level, the nuclear protein extracts of the *T. reesei* strains were also prepared and determined by LC–MS/MS in this study. More peptides of Xyr1 could be detected in QE2X than the other two strains, QCBX and QCDX (Additional file 3: Table S2). That is, the P*egl2*-driven *xyr1* overexpression, instead of the P*cbh1*/P*cdna1*-driven overexpression, significantly improved the amount of the Xyr1 protein in the nucleus. It is known that a phenomenon of “quelling” in filamentous fungi displays the characteristic feature of post-transcriptional gene silencing (PTGS), which functions by mediating mRNA degradation or translational suppression [41, 42]. There may be a “quelling” mechanism in *T. reesei* in which the excessive mRNA of *xyr1* causes translation repression, ultimately leading to a reduction in the amount of Xyr1 in the nucleus. Thus, it can be speculated that the transcript levels of *cbh1* and *cbh2* do not correspond to the transcript levels of *xyr1* to some extent, but strictly to its protein levels.

Meanwhile, Xyr1 has been implicated in chromatin remodeling with histone modification, which has a regulatory role on cellulase gene expression in *T. reesei* [43]. Mello-de-Sousa et al. have demonstrated that the deletion of *xyr1* could trigger chromatin compaction in the upstream regions of the cellulase genes (*cbh1* and *cbh2*) under inducing conditions, which suggests that Xyr1 is necessary for the chromatin opening of these regions [30]. Our results further confirmed this conclusion. The *cbh1* and *cbh2* promoters of the *xyr1*-overexpressing strains were more accessible compared to the parental strain (Fig. 4A, B). However, the chromatin packing pattern of *cbh1* and *cbh2* genes did not exactly follow the transcript level of the *xyr1* gene. Among the three strong promoters tested, the moderately strong one, i.e., the P*egl2*-driven overexpression of *xyr1* was more beneficial for the chromatin accessibility of cellulase-encoding genes, which resulted in the higher gene expression. Furthermore, we also confirmed that Xyr1 could influence the chromatin remodeling of *xyr1* itself. The opening of the native *xyr1* promoter is more pronounced in the *xyr1*-overexpressing strains than in the parental strain (Fig. 4C). Till et al. have reported the presence of Xyr1 regulatory element in the *xyr1* promoter, which is involved in the Xyr1 autoregulatory mechanism [44]. Thus, the increase of chromatin accessibility of the *xyr1* gene may be related to the above mechanism, which warrants further investigation.

BGL is the rate-limiting enzyme in the cellulose hydrolysis process, whose overexpression is beneficial for optimization of cellulase system [12]. However, the overexpression of *xyr1* is not adequate to mediate the high-level expression of BGL, which may be attributed to the presence of less Xyr1 binding sites (XBS) on the *bgl* promoter [4, 45]. In the parental strain QEB4, the introduction of *bgl* gene under control of the strong *cbh1* promoter containing more XBS makes the native Xyr1 a limiting step to further improve the expression of *bgl* [13]. In this study, the overexpression of *xyr1* in the strain QEB4 resulted in an increase in the production of BGL by a maximum of 1.8-fold (Fig. 6D). This result indicates that simultaneous engineering of transcription factors and cellulase genes could be a feasible strategy for improvement of cellulase production.

It is known that the efficiency of saccharification of lignocellulosic substrates is closely related to the performance of lignocellulases [11]. However, the composition of the enzyme complexes secreted by *T. reesei* may not be optimal for industrial applications due to the presence of certain enzymes in limiting amounts [9, 46]. Optimization of enzyme complex with different proportions of cellulase components and accessory proteins has been employed for highly efficient bioconversion of lignocellulosic biomass [12, 47]. In addition, lignocellulose is embedded in an amorphous matrix of cross-linked lignin and hemicellulose, which limits the cellulase accessibility to substrates [48, 49]. Especially, lignin is considered as the rate-limiting factor in the hydrolysis of lignocellulosic biomass as it impedes the access of cellulase to cellulose leading to non-productive binding [50, 51]. Hence, delignification of lignocellulosic biomass can improve the saccharification efficiency and saccharide yields [52, 53]. BSA and surfactants have also been demonstrated to prevent cellulase–lignin interactions to some extent [54, 55]. Furthermore, exogenous addition of Cip1 promoted the degradation of lignocellulosic substrates with high lignin [36]. Analysis of the extracellular proteins of QE2X by LC–MS/MS demonstrated the higher amounts of accessory proteins (Cip1, Cip2, Swo1, and LPMO) in QE2X than the parental strain QEB4 (Table 1), which altered the diversity of enzyme mixtures. Thus, the hydrolysis efficiency of ACR through the crude enzyme produced by QE2X had been greatly improved, showing the P*egl2*-driven *xyr1* overexpression could optimize the lignocellulolytic enzyme system by improving expression of accessory proteins.

## Conclusion

**Fig. 9:**
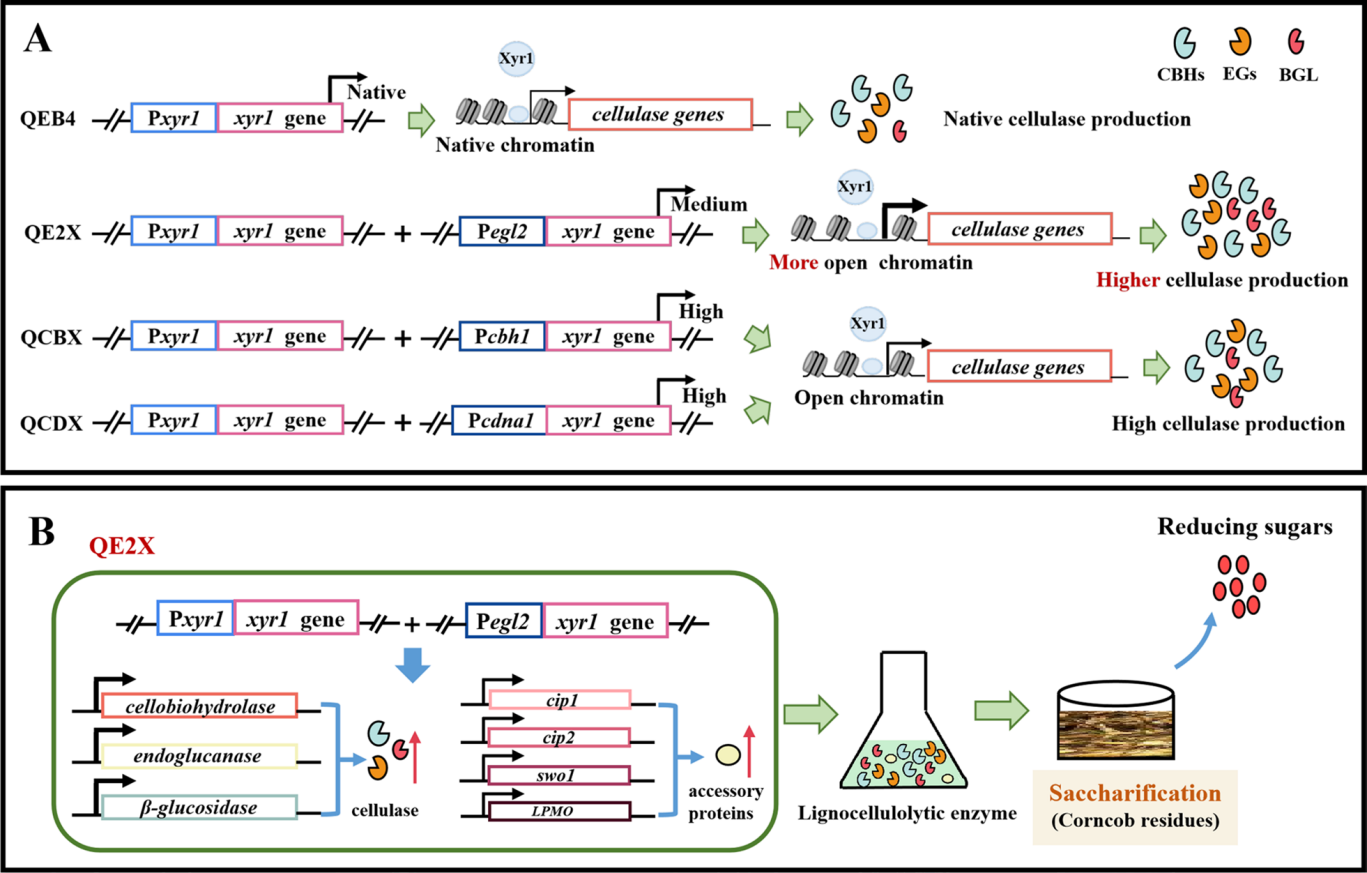
Schematic summary showing the proposed model for the dosage regulation of *xyr1* in *T. reesei*. **A** Compared to the strong *xyr1* overexpression with the *cbh1*/*cdna1* promoter, the moderate *xyr1* overexpression with the *egl2* promoter can result in more open chromatin and stronger expression of cellulase gene, thus significantly enhancing cellulase production. **B** The lignocellulolytic enzyme production and saccharification efficiency were significantly improved in *T. reesei* QE2X, because of the high expression of cellulase and accessory proteins

In the present study, we showed that the P*egl2*-driven *xyr1* overexpression significantly improved production of lignocellulolytic enzymes in *T. reesei* (Fig. 9). The total cellulase activity of QE2X was up to 8.4 IU/mL under inducing condition. The glucose release from ACR by the enzyme mixture of QE2X was improved by 57.2%. To the best of our knowledge, this is the first report regarding the roles of *xyr1* dosage level on cellulase expression in *T. reesei*. The significantly enhanced production of cellulase in QE2X is attributed to the moderate upregulation of transcription factor, with the underlying mechanisms require further exploration. In conclusion, this study demonstrates that selecting the promoter with proper dosage to drive the expression of key regulators is a feasible strategy to enhance lignocellulolytic enzyme production for the conversion of lignocellulose biomass.

## Materials and methods

### Strains, medium, and culture conditions

*T. reesei* QEB4, an EG2-BGL1 double overexpression strain [13], was used as the parental strain in this study. The strain QEB4 was constructed from the uracil auxotrophic strain QP4 whose *pyr4* gene was replaced with the resistance gene *hph*. The fungal strain and its derivatives were cultivated on potato dextrose agar (PDA) plates at 30 ℃ for 5–7 days to produce conidia, which were harvested, and then inoculated with approximately 10^6^ spores/mL into 50 mL of liquid minimal medium (MM) [56] before incubation for 36 h, 30 ℃ and 200 rpm. Then, equal amounts (0.4 g cell wet weight) of mycelia were transferred into 100 mL of the cellulase production medium (CPM) in 500 mL Erlenmeyer flasks. The CPM solution was composed as follows: 2% (*w/v*) Avicel, 0.1% CaCl_2_·2H_2_O, 0.5% (NH_4_)_2_SO_4_, 0.5% KH_2_PO_4_, 0.06% MgSO_4_·7H_2_O, and 2% corn steep liquor.

### Construction of the xyr1 overexpression strains

The *xyr1* overexpression cassettes were constructed with the double-joint PCR method [57]. The Phanta® Super-Fidelity DNA Polymerase (Vazyme Biotech Co., Ltd., Nanjing, China) was used for PCR amplification, and DNA fragments were purified using Gel Extraction Kit (Omega, USA). The primers were designed using the primer premier 5.0 software. Primer synthesis and DNA sequencing were performed at BGI Genomics (Qingdao, China). The primers used in this study are presented in Additional file 2: Table S1. The plasmid T-*ptrA* was used as the template to clone the *ptrA* expression cassette by PtrA-F/PtrA-R. The 5′- and 3′-flank fragments of *hph*, the three promoters (*egl2*, *cbh1,* and *cdna1*), and the encoding region and terminator of *xyr1* were amplified from the genomic DNA of *T. reesei* QEB4 using the primer pairs Hph-UF/Hph-UR, Hph-DF/Hph-DR, Egl2-F/Egl2-R, Cbh1-F/Cbh1-R, Cdna1-F/Cdna1-R, and Xyr1-F/Xyr1-R, respectively. Subsequently, the purified DNA fragments were fused together in 1:2:4:2:1 molar ratio of 5′-flanking region:*ptrA*: promoter:*xyr1*:3′-flanking region and were used as the template to amplify the final expression cassettes by Hph-cUF/Hph-cDR. The cassettes were transformed into the protoplasts of *T. reesei* QEB4 using the method described previously, respectively [56]. The strains were screened on MM agar plates containing 300 μg/mL pyrithiamine, and the purified candidate transformants were identified and named as QE2X, QCBX, and QCDX, respectively.

### RNA extraction and RT-qPCR analysis

The fungal mycelia were collected and flash-frozen in liquid nitrogen. Total RNA was extracted using the Trizol reagent (TaKaRa, Japan), and the RNA concentration was quantified by using a NanoDrop LiTE Spectrophotometer (THERMO SCIENTIFIC, United States). 1 μg RNA was reverse transcribed to cDNA using PrimeScript RT reagent Kit (TaKaRa, Japan) following the manufacturer’s protocol. RT-qPCR analysis was performed with SYBR Premix Ex Taq™ (TaKaRa, Japan) and the LigntCycler 480 system (Roche, Mannheim, Germany) using the primers listed in Additional file 2: Table S1. Three biological replicates were carried out for each sample. Transcript levels of target genes were analyzed according to the ΔΔCt method using the *actin* gene for normalization.

### Growth and secretion ability analysis

To analyze the growth morphology on plate, the parental and recombinant strains were pre-grown on MM agar plates and then equal amounts of mycelia were transferred in biological duplicates onto PDA plates or MM plates containing 2% glucose, 2% glycerol, or 2% lactose, respectively, and incubated at 30 ℃ for 2 days. Mycelia diameters were measured every 12 h for 2 days and recorded as representation of growth rates.

To determine the secretion ability of total cellulase, the equal amount of mycelia was inoculated on MCC plate containing 0.05% ball-milled Avicel, 0.02% peptone on the upper layer, and water agar on the bottom layer. After 5 days incubation, the double-layer MCC plate with hydrolysis zones was measured and recorded by photographs. In addition, the secretion ability of EGs and BGL1 in recombinant strains was assayed by the CMC-esculin plates (1% CMC-Na, 0.3% esculin, 0.05% ferric citrate and 2% agar) and the CMC plates (1% CMC-Na, 0.1% yeast extract and 2% agar), respectively.

### Enzyme activity and protein assay

The activities of FPase (FPA) and EGs were determined by measuring the released reducing sugar with filter paper and CMC-Na as substrates, respectively. Determination of FPA was performed in a 2 mL reaction system containing 500 μL of the appropriately diluted culture supernatant, 1.5 mL of 50 mM citrate buffer (pH 4.8), and 0.05 g of Whatman No.1 filter paper. The EGs activity assay was carried out at 50 ℃ in a 2 mL reaction system including 500 μL of the suitably diluted supernatant and 1.5 mL of 0.5% CMC sodium in 50 mM citrate buffer (PH 4.8). The DNS method was used to measure the amount of released reducing sugars [58]. The CBHs and BGL activities were determined by measuring the amount of released p-nitrophenol using p-nitrophenyl-D-cellobioside (pNPC, Sigma, USA) and p-nitrophenyl-β-D-glucopyranoside (pNPG, Sigma, USA) as the substrates, respectively. The assays were performed in 200 μL reaction mixtures containing 50 μL of the diluted supernatant, 50 μL of the respective substrate, and 100 μL of 50 mM citrate buffer (pH 4.8) and then incubated at 50 ℃ for 30 min. One unit (U) of enzyme activity was defined as the amount of enzymes liberating 1 µmoL reducing sugars (FPA, EGs) or p-nitrophenol (CBHs, BGL1) per minute under the test conditions. Total extracellular proteins were assayed as previously described by Ellilä et al*.* [4]. Briefly, proteins were precipitated with ice-cold acetone and the concentration was quantified using the Bradford Kit (Sangon Biotech, Shanghai, China) with bovine serum albumin (BSA) as a standard. To facilitate analyzing the amount of the Xyr1 protein, total nuclear protein extracts were prepared from the *T. reesei* strains cultured on 2% (*w/v*) Avicel for 3 days. And the extraction experiment was performed using the Filamentous fungi Nuclear Protein Extraction Kit (BestBio, Shanghai, China) according to the manufacturer’s instructions.

### Saccharification of the pretreated corncob residues

For the saccharification assay, ACR and DCR were applied as substrates in the saccharification process. ACR consists of 62.6% cellulose, 2.4% hemicellulose, 17.7% lignin, and 6.8% ash, while DCR contains 65.7% cellulose, 1.8% hemicellulose, 3.2% lignin, and 5.9% ash [59]. The crude enzyme preparations were placed in 30 mL reaction volume in 100 mL flasks containing 50 mM citrate buffer (pH 4.8), 1.5 g substrate, and 30 μL Proclin 300 preservative, and the reaction mixture was incubated at 200 rpm, 50 ℃. Enzyme loading was adjusted to the same FPA (10 FPU/g substrate). Glucose released was detected by the SBA-40C biological sensor analyzer (BISAS, Shandong, China) after incubation for 48 h.

### Southern blot analysis

The specific probe was amplified from the genome of *T. reesei* QEB4 using the primer pair Hph-pF/Hph-pR. Chromosomal DNA of the parental and recombinant strains was digested thoroughly by *Mph1103I* and then hybridized by the probe. Finally, the probe-hybridized DNA fragments were detected and visualized using a DIG-High Prime DNA Labeling and Detection Starter Kit I (Roche, Germany) according to the manufacturer’s instructions.

### Chromatin accessibility real‑time PCR (CHART‑PCR) assays

CHART-PCR assay were performed according to a previously described protocol [60]. Briefly, mycelia were collected after 48 h of induction, filtered, and ground in liquid nitrogen. Subsequently, 0.1 g powder was incubated with 10 μL RNase-free DNase I (TaKaRa Biotechnology) for 5 min at 37 ℃ in 1 mL of nuclease digestion buffer (250 mM sucrose, 0.05 mM CaCl_2_, 3 mM MgCl_2_, 15 mM NaCl, 60 mM KCl, 5 mM DTT, and 15 mM pH 7.5 Tris–HCl). The reaction was terminated by adding 100 μL of termination buffer (20 mM EDTA and 2% SDS). Then, two rounds of phenol–chloroform extraction and one round of chloroform extraction were followed for protein extraction. The supernatant was treated with RNase A (10 μg/mL) at 37 ℃ for 15 min and precipitated with 0.3 M NaAc and two volumes of ethanol. The DNA obtained were dissolved in 30 μL of double-distilled water. A negative control reaction without DNase I was included. Quantitative PCR analysis on the DNase I-treatment samples was performed to measure the relative abundance of target regions. Each sample was prepared in triplicate. The chromatin accessibility index (CAI) was defined as CAI = Dc/Ds, where Dc is the amount of intact DNA detected for the promoter regions of *actin* gene and Ds is the amount of intact DNA detected for each target region. The amount of intact input DNA for each sample was calculated by comparing the thresholds of the PCR amplification plots with the standard curve produced for each primer set using serial dilutions of undigested genomic DNA. All primer sequences are provided in Additional file 2: Table S1.

### LC–MS/MS

LC–MS/MS analyses were performed according to the method described previously with appropriate modifications [61]. The fermentation supernatants of three biological replicates were mixed in equal proportions and then subjected to the measurement. The sample (100 μg of protein, measured by the Bradford method) was denatured in an equal volume of 8 M urea and reduced in 5 mM DTT at 56 ℃ for 30 min. The samples were then alkylated in 14 mM iodoacetamide (IAA) for 1 h in darkness. The unreacted IAA was quenched by the addition of 5 mM DTT for 15 min. The samples were digested by adding trypsin (Sigma, USA) with 1 mM CaCl_2_ as a co-factor at 37 ℃ for 12 h, and reaction was terminated by adjusting to pH 2 with trifluoroacetic acid. The digested oligopeptides were desalted on C18 Sep-Pak cartridges (Waters Associates, USA) and were further dissolved in 0.1% formic acid and then were subjected to nanoelectrospray ionization, followed by analysis in LTQ Orbitrap Velos Pro (Thermo Scientific, USA) coupled with HPLC system. The Orbitrap resolution was set at 70,000, and data from LC–MS/MS analysis were searched by proteome discovered software 1.4 (Thermo Fisher Scientific). Sequences were mapped based on the reference genome of *T. reesei*, acquired from the JGI Genome Portal (https://mycocosm.jgi.doe.gov/Trire2/Trire2.home.html). Furthermore, the relative protein abundance was characterized by peptide spectrum matches (PSMs), which could correlate linearly with the protein abundance [62].

## Supplementary Information

**Additional file 1: Figure S1.** Growth assay of *T. reesei* QEB4 and the *xyr1* overexpression strains. (A) Colonial phenotypes of the parental strain and three recombinant strains on MM with 2% glucose, 2% glycerol, and 2% lactose or on PDA. Plates were incubated at 30℃ and photos were taken at 48 h. Growth rates of hyphae were determined after cultivation on the MM plates with the following carbon sources: glucose (B), glycerol (C), and lactose (D), and on the PDA plates (E) at 30°C for 48 h. Results are means of three biological replicates and error bars indicate ± SD

**Additional file 2: Table S1.** Primers used in this study

**Additional file 3: Table S2.** The Xyr1 protein identified from the nucleus of *T. reesei* by LC-MS/MS

## Data Availability

The datasets used and/or analyzed during the current study are available from the corresponding author on reasonable request.

## Abbreviations

CBHs: Cellobiohydrolases
EGs: Endoglucanases
BGL: β-Glucosidase
MM: Minimal medium
CPM: Cellulase production medium
CMC-Na: Sodium carboxymethy cellulose
pNPG: 4-Nitrophenyl-β-D-glucopyranoside
pNPC: P-nitrophenyl-β-D-cellobioside
FPA: Filter paper activity
BSA: Bovine serum albumin
ACR: Acid-pretreated corncob residues
DCR: Delignified corncob residues
CAI: Chromatin accessibility index
PSMs: Peptide spectrum matches
XBS: Xyr1 binding sites
PTGS: Post-transcriptional gene silencing
