# Supplementary material for: Tailoring the expression of Xyr1 leads to efficient production of lignocellulolytic enzymes in Trichoderma reesei for improved saccharification of corncob residues
Source: Biotechnol Biofuels Bioprod. 2022 Dec 17;15:142. doi: 10.1186/s13068-022-02240-9 (PMC9759857; doi:10.1186/s13068-022-02240-9)
Supplement: Supplementary file 1 — Additional file 1: Figure S1. Growth assay of T. reesei QEB4 and the xyr1 overexpression strains. (A) Colonial phenotypes of the parental strain and three recombinant strains on MM with 2% glucose, 2% glycerol, and 2% lactose or on PDA. Plates were incubated at 30℃ and photos were taken at 48 h. Growth rates of hyphae were determined after cultivation on the MM plates with the following carbon sources: glucose (B), glycerol (C), and lactose (D), and on the PDA plates (E) at 30°C for 48 h. Results are means of three biological replicates and error bars indicate ± SD [file 13068_2022_2240_MOESM1_ESM.docx]

###
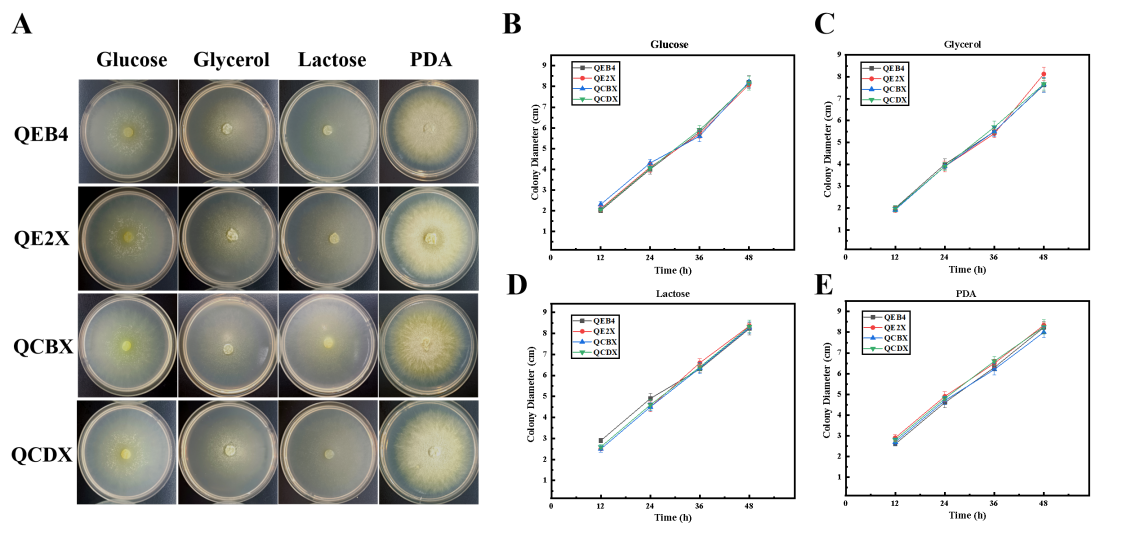


**Figure S1.** Growth assay of *T. reesei* QEB4 and the *xyr1* overexpression strains. (A) Colonial phenotypes of the parental strain and three recombinant strains on MM with 2% glucose, 2% glycerol, and 2% lactose or on PDA. Plates were incubated at 30℃ and photos were taken at 48 h. Growth rates of hyphae were determined after cultivation on the MM plates wtih the following carbon sources: glucose (B), glycerol (C) and lactose (D), and on the PDA plates (E) at 30°C for 48 h. Results are means of three biological replicates and error bars indicate ± SD.
