# Supplementary material for: Tailoring the expression of Xyr1 leads to efficient production of lignocellulolytic enzymes in Trichoderma reesei for improved saccharification of corncob residues
Source: Biotechnol Biofuels Bioprod. 2022 Dec 17;15:142. doi: 10.1186/s13068-022-02240-9 (PMC9759857; doi:10.1186/s13068-022-02240-9)
Supplement: Supplementary file 2 — Additional file 2: Table S1. Primers used in this study [file 13068_2022_2240_MOESM2_ESM.docx]

**Table S2**

Primers used in this study.

| Primers | Nucleotide sequence（5’ to 3’） | Employment |
| --- | --- | --- |
| Hph-UF | GCTGTTCTCCAAGGCGTCA | Strain construction |
| Hph-UR (PtrA) | AACAAAGATGCAAGAGCGGGGAGCCGAGAGGGTAGTAATG | Strain construction |
| Hph-DF | AGAAAGGCATTTAGCAAGAAGG | Strain construction |
| Hph-DR | TTCAGGGCGAAGCTGTCC | Strain construction |
| Hph-cUF | CAGGTGGATCAGCAAAGTTG | Strain construction |
| Hph-cDR | GTAGCCGTGGCGGTCATT | Strain construction |
| Egl2-F | AAACACCTCGCTCCAGTGC | Strain construction |
| Egl2-R | TGTCGATGACGGGGAGATAT | Strain construction |
| Cbh1-F | AAAGCGTTCCGTCGCAGTAG | Strain construction |
| Cbh1-R | GATGCGCAGTCCGCGGTT | Strain construction |
| Cdna1-F | CAGACAATGATGGTAGCAGC | Strain construction |
| Cdna1-R | GATCAATCCAACAACTTCTCTC | Strain construction |
| Xyr1-F (Egl2) | ATATCTCCCCGTCATCGACAATGTTGTCCAATCCTCTCCGT | Strain construction |
| Xyr1-F (Cbh1) | AACCGCGGACTGCGCATCATGTTGTCCAATCCTCTCCGT | Strain construction |
| Xyr1-F (Cdna1) | GAGAGAAGTTGTTGGATTGATCATGTTGTCCAATCCTCTCCGT | Strain construction |
| Xyr1-R (Hph) | CCTTCTTGCTAAATGCCTTTCTCTGCACGCATCATAGAATCG | Strain construction |
| PtrA-F | CCGCTCTTGCATCTTTGTT | Strain construction |
| PtrA-R (Egl2) | TGAACCGATTGCTGCGATCCCCAGGCTTTACACTTTAT | Strain construction |
| PtrA-R (Cbh1) | CTACTGCGACGGAACGCTTTCCCCAGGCTTTACACTTTAT | Strain construction |
| PtrA-R (Cdna1) | GCTGCTACCATCATTGTCTGCCCCAGGCTTTACACTTTAT | Strain construction |
| Hph-pF | AGGGAGGACAAACGGAGCT | Probe |
| Hph-pR | ATATCGCCTTGGGTTGCC | Probe |
| Actin-qF | CCCAAGTCCAACCGTGAGA | RT-qPCR for *actin* |
| Actin-qR | CAATGGCGTGAGGAAGAGC | RT-qPCR for *actin* |
| Xyr1-qF | TCTTCTACGGCGTCTATCTCC | RT-qPCR for *xyr1* |
| Xyr1-qR | GTGTGCCCTAACAATGGTCTC | RT-qPCR for *xyr1* |
| Cbh1-qF | GCGGCATGGTTCTGGTCA | RT-qPCR for *cbh1* |
| Cbh1-qR | TCGTTTGTCGGGTAGGTGGA | RT-qPCR for *cbh1* |
| Cbh2-qF | CTGGTCCAACGCCTTCTTCA | RT-qPCR for *cbh2* |
| Cbh2-qR | GACCCAGACAAACGAATCCAG | RT-qPCR for *cbh2* |
| Egl1-qF | CGGCTACAAAAGCTACTACG | RT-qPCR for *egl1* |
| Egl1-qR | CTGGTACTTGCGGGTGAT | RT-qPCR for *egl1* |
| Egl2-qF | ACGAGCCTTTGGTCGCAGTT | RT-qPCR for *egl2* |
| Egl2-qR | GGCAGCCCAGGTGTTGATGT | RT-qPCR for *egl2* |
| Bgl1-qF | AGTGACAGCTTCAGCGAG | RT-qPCR for *bgl1* |
| Bgl1-qR | GGAGAGGCGTGAGTAGTTG | RT-qPCR for *bgl1* |
| Cre1-qF | GCAGCACAATACGACTCCG | RT-qPCR for *cre1* |
| Cre1-qR | GGCTAATGATGTCGGTAAGTGA | RT-qPCR for *cre1* |
| Ace1-qF | ACCAAGACCAACGGCAAGA | RT-qPCR for *ace1* |
| Ace1-qR | CGTGGAGGAAGGCGTAGACA | RT-qPCR for *ace1* |
| Ace2-qF | GCCTCAATGCTGCTCTCTGTT | RT-qPCR for *ace2* |
| Ace2-qR | GACGAACGACCTTTGCTTCTCT | RT-qPCR for *ace2* |
| Ace3-qF | ATTGTGCGAGACATGCTGAG | RT-qPCR for *ace3* |
| Ace3-qR | GATGGCCAGCAAACTAGCTC | RT-qPCR for *ace3* |
| Vib1-qF | TGACCTGCTACCGAAGAAACC | RT-qPCR for *vib1* |
| Vib1-qR | CCACGGGATGACAATAAGACG | RT-qPCR for *vib1* |
| Ctf1-qF | TCAACCAAAAGCCAAAGGAG | RT-qPCR for *ctf1* |
| Ctf1-qR | GGGTCAAAGTCGGTGTGTG | RT-qPCR for *ctf1* |
| Pdi1-qF | GTTGTCGTTGCCCACTCTTAC | RT-qPCR for *pdi1* |
| Pdi1-qR | AGTCGCTCTTGGCATACAGG | RT-qPCR for *pdi1* |
| Bip1-qF | GATGCCAACGGTATCCTCA | RT-qPCR for *bip1* |
| Bip1-qR | TGCGGTCAATCTCCTCCT | RT-qPCR for *bip1* |
| Hrd1-qF | CCCAATGATGCCAAACTG | RT-qPCR for *hrd1* |
| Hrd1-qR | TGCTGCTTTCAGGTGGAG | RT-qPCR for *hrd1* |
| Der1-qF | CCTCGTTTACATTTGGTCTCG | RT-qPCR for *der1* |
| Der1-qR | GCCCATGATCTCATCCCTC | RT-qPCR for *der1* |
| Cip1-qF | TGTCAACTTTGGCTGGGAG | RT-qPCR for *cip1* |
| Cip1-qR | CGGAATGGTGGTGCTTGG | RT-qPCR for *cip1* |
| Cip2-qF | CGCTTGCTGCCTTGATAG | RT-qPCR for *cip2* |
| Cip2-qR | GTTGCGATTGCTGGTTTG | RT-qPCR for *cip2* |
| Swo1-qF | CTCACGGCAGTCAACACC | RT-qPCR for *swo1* |
| Swo1-qR | TCTTGTGGGCGGCTACTC | RT-qPCR for *swo1* |
| Cel61a-qF | CCGCTCAACTACATCATCCC | RT-qPCR for *cel61a* |
| Cel61a-qR | TGTCGTGGTTCTGCTGGTC | RT-qPCR for *cel61a* |
| Actin-ctF | CTTCCCTCCTTTCCTCCCCCTCCA | *actin* CHART |
| Actin-ctR | GCGACAGGTGCACGTACCCTCCATT | *actin* CHART |
| Cbh1-ctF | GGATCGAACACACTGCTGCCTTTAC | *cbh1* CHART |
| Cbh1-ctR | GGTTTCTGTGCCTCAAAAGATGGTG | *cbh1* CHART |
| Cbh2-ctF | GCAGCGCAACACTACACGCAACAT | *cbh2* CHART |
| Cbh2-ctR | TGCGCCTCATACAGGGTCACAGTCC | *cbh2* CHART |
| Xyr1-ctF | CCGACAGCAGCAGTAGTCAGGTTTT | *xyr1* CHART |
| Xyr1-ctR | TAGGCAGAATAGCGACGGAGAGGAT | *xyr1* CHART |
