# Supplementary material for: Tailoring the expression of Xyr1 leads to efficient production of lignocellulolytic enzymes in Trichoderma reesei for improved saccharification of corncob residues
Source: Biotechnol Biofuels Bioprod. 2022 Dec 17;15:142. doi: 10.1186/s13068-022-02240-9 (PMC9759857; doi:10.1186/s13068-022-02240-9)
Supplement: Supplementary file 3 — Additional file 3: Table S2. The Xyr1 protein identified from the nucleus of T. reesei by LC-MS/MS [file 13068_2022_2240_MOESM3_ESM.docx]

**Table S2.** The Xyr1 protein identified from the nucleus of *T. reesei* by LC-MS/MS.

| Protein ID | Name | Strains | | | |
| --- | --- | --- | --- | --- | --- |
|  |  | QEB4 | QE2X | QCBX | QCDX |
| 122208 | Xyr1 | Not detected | 7 | 3 | 2 |
